# Comparison of large‐scale citizen science data and long‐term study data for phenology modeling

**DOI:** 10.1002/ecy.2568

**Published:** 2018-12-24

**Authors:** Shawn D. Taylor, Joan M. Meiners, Kristina Riemer, Michael C. Orr, Ethan P. White

**Affiliations:** ^1^ School of Natural Resources and Environment University of Florida PO Box 116455 Gainesville Florida 32611 USA; ^2^ Department of Wildlife Ecology and Conservation University of Florida PO Box 110430 Gainesville Florida 32611 USA; ^3^ Key Laboratory of Zoological Systematics and Evolution Institute of Zoology Chinese Academy of Sciences Beijing 100101 China; ^4^ Informatics Institute University of Florida PO Box 115585 Gainesville Florida 32611 USA

**Keywords:** budburst, data integration, flowering, forecasting, Long Term Ecological Research, scale, USA National Phenology Network

## Abstract

Large‐scale observational data from citizen science efforts are becoming increasingly common in ecology, and researchers often choose between these and data from intensive local‐scale studies for their analyses. This choice has potential trade‐offs related to spatial scale, observer variance, and interannual variability. Here we explored this issue with phenology by comparing models built using data from the large‐scale, citizen science USA National Phenology Network (USA‐NPN) effort with models built using data from more intensive studies at Long Term Ecological Research (LTER) sites. We built statistical and process based phenology models for species common to each data set. From these models, we compared parameter estimates, estimates of phenological events, and out‐of‐sample errors between models derived from both USA‐NPN and LTER data. We found that model parameter estimates for the same species were most similar between the two data sets when using simple models, but parameter estimates varied widely as model complexity increased. Despite this, estimates for the date of phenological events and out‐of‐sample errors were similar, regardless of the model chosen. Predictions for USA‐NPN data had the lowest error when using models built from the USA‐NPN data, while LTER predictions were best made using LTER‐derived models, confirming that models perform best when applied at the same scale they were built. This difference in the cross‐scale model comparison is likely due to variation in phenological requirements within species. Models using the USA‐NPN data set can integrate parameters over a large spatial scale while those using an LTER data set can only estimate parameters for a single location. Accordingly, the choice of data set depends on the research question. Inferences about species‐specific phenological requirements are best made with LTER data, and if USA‐NPN or similar data are all that is available, then analyses should be limited to simple models. Large‐scale predictive modeling is best done with the larger‐scale USA‐NPN data, which has high spatial representation and a large regional species pool. LTER data sets, on the other hand, have high site fidelity and thus characterize inter‐annual variability extremely well. Future research aimed at forecasting phenology events for particular species over larger scales should develop models that integrate the strengths of both data sets.

## Introduction

Plant phenology, the timing of recurring biological events such as flowering, plays an important role in ecological research extending from local to global scales (Cleland et al. 2007, Richardson et al. 2013, Tang et al. 2016). At large scales the timing of spring leaf out and fall senescence influence the carbon budget of earth system models, which has implications for correctly accounting for biosphere–atmosphere feedbacks in long‐term climate forecasts (Richardson et al. 2012). At smaller scales, species‐specific responses to temperature and precipitation can alter flower communities (Diez et al. 2012, CaraDonna et al. 2014, Theobald et al. 2017) and affect the abundance and richness of both pollinators (Ogilvie and Forrest 2017, Ogilvie et al. 2017) and organisms at higher trophic levels (Tylianakis et al. 2008). Plant phenology models that are robust at multiple ecological scales, or deemed appropriate for a particular scale, are needed to better understand and forecast the timing of key biological events.

Many plant phenology studies use intensively collected data sets from a single location over a long time‐period by a single research group (Cook et al. 2012, Wolkovich et al. 2012, Iler et al. 2013, Roberts et al. 2015). These data sets have regular sampling and large numbers of samples over long periods of time. As a result, the biological and climatic variability at that site is well represented. It is common for phenology models built with observations from a single site to not transfer well to other sites (García‐Mozo et al. 2008, Xu and Chen 2013, Olsson and Jönsson 2014, Basler 2016). This lack of transferability can be driven by plasticity in phenology requirements, local adaptation, microclimates, or differences in plant age or population density (Kramer 1995, Diez et al. 2012). For these reasons, data from a single location are not adequate for larger scale phenology modeling. Accurately forecasting phenology at larger scales will require models that account for the full range of variation across a species’ range (Richardson et al. 2013, Tang et al. 2016, Chuine and Régnière 2017), which will necessitate the use of data sources beyond traditional single‐site studies.

Data from citizen science projects are becoming increasingly important for ecological research (Kelling et al. 2009, Dickinson et al. 2010, Tulloch et al. 2013). Because these data are often collected by large numbers of volunteers, it is possible to gather data at much larger scales than with individual research teams. A relatively new citizen science project started in 2009, Nature's Notebook run by The USA National Phenology Network (USA‐NPN), collects phenology observations from volunteers throughout the United States and makes the data openly available (Schwartz et al. 2012). Data from this project have already been used to study variation in oak phenology at a continental scale (Gerst et al. 2017), develop large‐scale community phenology models (Melaas et al. 2016), and forecast long‐term phenology trends (Jeong et al. 2013). Large‐scale data sets from China and Europe have already contributed considerably to phenological research (Xu and Chen 2013, Olsson and Jönsson 2014, Basler 2016, Zhang et al. 2017), and the USA‐NPN data set has the potential to meet these needs for North American plant species and communities. However, the features that allow citizen science projects to collect data at large scales can also introduce spatial biases toward cities and easily‐accessible areas, and variation in sampling effort and observer skill (Dickinson et al. 2010). With thousands of participants, the potential for variation among observers in their determination of species identification and dating of phenological events is high. While volunteers have been shown to be accurate at distinguishing different leaf and flower stages for plants (Fuccillo et al. 2015) and can have high agreement on abundance estimates (Feldman et al. 2018), contributions to USA‐NPN are sometimes made sporadically across seasons, years, and locations. This means that the quantity and quality of data at a specific site will typically be more variable for citizen science efforts than for intensive, long‐term studies.

In order to accurately model and forecast phenology, it is important to understand how the strengths and weaknesses of intensive local studies and large‐scale citizen science projects influence both our inferences about biological processes driving phenology (e.g., warming requirements for a specific plant) and our ability to predict future phenology events (e.g., forecasting when flowering or leaf out occurs). Here, we fit a suite of plant phenology models for the budburst and first‐flowering phenophases of 24 plant species to data from both the USA‐NPN and a set of intensive long‐term studies from the Long Term Ecological Research (LTER) network. We compare the resulting models based on both inference about models and parameters and predictions for unobserved events. We then use this comparison to assess the best methods for both local‐ and large‐scale phenology modeling and to point the way forward for integrating large‐scale and local‐scale data to determine the best possible models across scales.

## Methods

### Data sets

**Figure 1:**
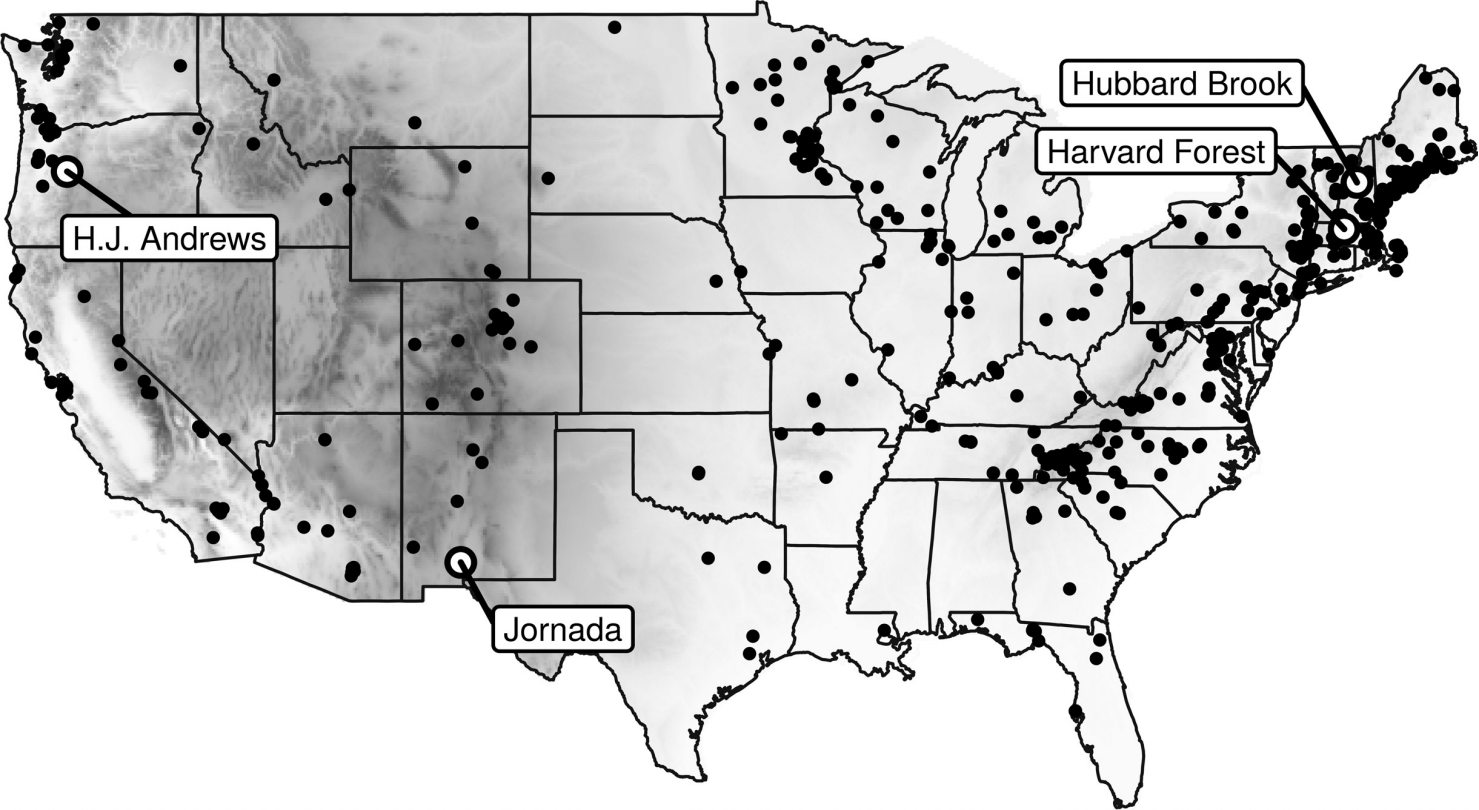
Locations of USA National Phenology Network sites used (black points) and Long Term Ecological Research sites (LTER; labeled circles), with gray scale showing elevation.

The USA National Phenology Network (USA‐NPN) protocol uses status‐based monitoring, where via a phone app or web based interface observers answer “yes,” “no,” or “unsure” when asked if an individual plant has a specific phenophase present (Denny et al. 2014). Phenophases refer to specific phases in the annual cycle of a plant, such as the presence of emerging leaves, flowers, fruit, or senescing leaves. Sites in the USA‐NPN data sets are located across the United States and generally clustered around populated areas (Fig. 1). To represent long‐term, intensive, phenology studies we used four data sets from North America representing three major ecosystem types (Table 1, Fig. 1). All four long‐term studies are located in the United States and are part of the Long Term Ecological Research network (LTER). The Harvard Forest and Hubbard Brook Long Term Experimental Forest are located in the northeastern United States and are dominated by deciduous broadleaf species. The H.J. Andrews Experimental Forest is a coniferous forest in the coastal range of the western United States The Jornada Experimental Range is in the Chihuahua desert of the southwestern U.S.

**Table 1:** LTER data sets used in the analysis

Data set name	Habitat	Phenological event (no. species)	Source
Harvard Forest	Northeast deciduous forest	Budburst (17) Flowering (7)	O'Keefe (2015)
Jornada Experimental Range	Chihuahuan Desert	Flowering (2)	
H.J. Andrews Experimental Forest	Northwest wet coniferous forest	Budburst (5) Flowering (4)	Schulze (2017)
Hubbard Brook	Northeast deciduous forest	Budburst (3)	Bailey (2018)

We downloaded all USA‐NPN observations from 2009, when collections began, to 2016 for the following phenophases: Breaking Leaf Buds, Breaking Needle Buds, Emerging Needles, and Open Flowers (USA National Phenology Network, 2017). The first three phenophases apply to the “leaf out” phase for deciduous broadleafs, evergreen conifers, and pines, respectively. The “Open Flowers” phenophase refers to fully open flowers and applies to all angiosperms. Hereafter, we will refer to these as either “Flowers” for the Open Flower phenophase, or “Budburst” for all other phenophases. We subset the USA‐NPN observations similar to methods outlined in Crimmins et al. (2017). First, “yes” observations for individual plants were kept only if they were preceded by a “no” observation within 30 d. Observations for “Budburst” that were past day of year (DOY) 172, and for “Flowers” that were past DOY 213 were dropped to minimize any influence from outliers. We inferred the observed DOY of each phenophase as the midpoint between each “yes” observation and the preceding “no” observation. Finally, only species that had >30 total observations were kept. Crimmins et al. (2017) only kept observations that were preceded by a “no” within 15 d, and also grouped multiple individuals at single sites to a single observation. We used 30 d to allow for a greater number of species to be compared. We tested the sensitivity of this choice by also performing the analysis using a 15‐d cutoff. We chose not to group multiple individuals at a single site to better incorporate intra‐site variability.

In the LTER data sets observation metrics varied widely due to different protocols. To match the USA‐NPN data we converted all metrics to binary “yes” and “no” observations for each phenophase (see Appendix S1 for details). Three of the LTER data sets (Hubbard Brook, Harvard Forest, and H.J. Andrews) had a sampling frequency of 3–7 d during the growing season. The Jornada data set had a sampling frequency of 30 d. As with the USA‐NPN data, we inferred the date for each phenophase as the midpoint between the first “yes” observation and most recent “no” observation, and only kept species and phenophase combinations that had at least 30 total observations. After data processing there were 38 species and phenophase combinations (with 24 unique species) common to both the USA‐NPN and LTER data sets to use in the analysis (Table 1; Appendix S2: Table S1). Using a 15 d cutoff in the USA‐NPN data set resulted in 35 unique combinations with 23 species.

### Models

It is common to fit multiple plant phenology models to find the one that best represents a specific species and phenophase (Chuine et al. 2013). For each of the 38 species and phenophase combinations in the five data sets (USA‐NPN and four LTER data sets), we fit eight phenology models (Table 2). The Naive model uses the mean DOY from prior observations as the estimated DOY. The Linear model uses a regression with the mean spring (1 January–31 March) temperature as the independent variable and DOY as the response variable. For the six remaining models, the general form is based on the idea that a phenological event will occur once sufficient thermal forcing units, $F^{*}$, accumulate from a particular start day of the year ($t_{1}$). Forcing units are a transformation of the daily mean temperature and are calculated differently for each model (Table 2). The start day can either be estimated or fixed. For the Growing Degree Day (GDD) model, forcing units are the total degrees above the threshold $T_{\mathrm{base}}$ (Réaumur 1735, Wang 1960, Hunter and Lechowicz 1992). The Fixed GDD model uses the same form but has fixed values for start day ($t_{1}$ = 1 January) and temperature threshold ($T_{\mathrm{base}}$ = 0°C). The Alternating model has a variable number of required forcing units defined as a function of the total number of days below 0°C since 1 January (number of chill days; NCD). The Uniforc model is like the GDD model but with the forcing units transformed via a sigmoid function (Chuine 2000).

**Table 2:** Phenology models used in the analysis

Name	DOY estimator	Forcing equations	Total parameters	Source
Naive	$\bar{\text{DOY}}$	–	1	–
Linear	$\text{DOY}=\beta_{1}+\beta_{2}T_{\mathrm{mean}}$	–	2	–
GDD	$\sum_{t=t_{1}}^{\mathrm{DOY}}R_{f}\left(T_{i}\right)\geq F^{*}$	$R_{f}\left(T_{i}\right)=\max\left(T_{i}-T_{\mathrm{base}},0\right)$	3	Réaumur (1735), Wang (1960), Hunter and Lechowicz (1992)
Fixed GDD	$\sum_{t=1}^{\mathrm{DOY}}R_{f}\left(T_{i}\right)\geq F^{*}$	$R_{f}\left(T_{i}\right)=\max\left(T_{i},0\right)$	1	Réaumur (1735), Wang (1960), Hunter and Lechowicz (1992)
Alternating	$\sum_{t=1}^{\mathrm{DOY}}R_{f}\left(T_{i}\right)\geq a+be^{\mathrm{cNCD}(t)}$	$R_{f}\left(T_{i}\right)=\max\left(T_{i}-5,0\right)$	3	Cannell and Smith (1983)
Uniforc	$\sum_{t=t_{1}}^{\mathrm{DOY}}R_{f}\left(T_{i}\right)\geq F^{*}$	$R_{f}\left(T_{i}\right)=\frac{1}{1+e^{b(T_{i}-c)}}$	4	Chuine (2000)
M1	$\sum_{t=t_{1}}^{\mathrm{DOY}}R_{f}\left(T_{i}\right)\geq {\left(\frac{L_{i}}{24}\right)}^{k}F^{*}$	$R_{f}\left(T_{i}\right)=\max\left(T_{i}-T_{\mathrm{base}},5\right)$	4	Blümel and Chmielewski (2012)
MSB	$\sum_{t=1}^{\mathrm{DOY}}R_{f}\left(T_{i}\right)\geq a+be^{{\mathrm{cNCD}}_{i}}+dT_{\mathrm{mean}}$	$R_{f}\left(T_{i}\right)=\max\left(T_{i}-5,0\right)$	4	Jeong et al. (2013)

*Notes*

For all models, except the Naive and Linear models, the daily mean temperature $T_{i}$ is first transformed via the specified forcing equation. The cumulative sum of forcing is then calculated from a specific start date (either DOY=1 or using the fitted parameter $t_{1}$). The phenological event is estimated as the DOY in which cumulative forcing is greater than or equal to the specified total required forcing (either $F^{*}$ or the specified equation). Parameters for each model are as follows: for the Naive model $\bar{\text{DOY}}$ is the mean day of year of a phenological event; for the Linear model, $\beta_{1}$ and $\beta_{2}$ are the intercept and slope, respectively, and $T_{\mathrm{mean}}$ is the average daily temperature between 1 January and 31 March; for the GDD model $F^{*}$ is the total accumulated forcing required, $t_{1}$ is the start date of forcing accumulation, and $T_{\mathrm{base}}$ is the threshold daily mean temperature above which forcing accumulates; for the Fixed GDD model, $F^{*}$ is the total accumulated forcing required; for the Alternating model, NCD is the number of chill days (daily mean temperature below 0°C) from DOY=0 to the DOY of the phenological event, a, b, and c are the three fitted model coefficients; for the Uniforc model, $F^{*}$ is the total accumulated forcing required, $t_{1}$ is the start date of forcing accumulation, and b and c are two additional fitted parameters that define the sigmoid function; the M1 model is the same as the GDD model, but with the additional fitted parameter k that adjusts the total forcing accumulation according to day length; the MSB model is the same as the Alternating model, but with the additional fitted parameter d to correct the model according to mean spring temperature.

We also fit two models that attempt to capture spatial variation in phenological requirements. The first spatial model, M1, is an extension of the GDD model which adds a correction in the required forcing using the photoperiod (*L*, Blümel and Chmielewski 2012). The second, the Macroscale Species‐specific Budburst model (MSB), uses the mean spring temperature as a linear correction on the total forcing required in the Alternating model (Jeong et al. 2013). Since there is little to no spatial variation in the LTER data sets, we fit the two spatial models to data from the USA‐NPN only. We compared the resulting parameters, estimates, and errors for the USA‐NPN derived M1 and MSB models to their non‐spatial analogs (the GDD and Alternating models, respectively) for each species and phenophase in the LTER data.

We extracted corresponding daily mean temperature for all USA‐NPN and LTER observations from the gridded PRISM data set using the latitude and longitude of the site associated with each observation (PRISM Climate Group, 2004). We parameterized all models using differential evolution to minimize the root mean square error (RMSE) of the estimated DOY of the phenological event. Differential evolution is a global optimization algorithm that uses a population of randomly initialized models to find the set of parameters that minimize the RMSE (Storn and Price 1997). Confidence intervals for parameters were obtained by bootstrapping, in which individual models were refit 250 times using a random sample, with replacement, of the data. We made predictions by taking the mean DOY estimated from the 250 bootstrapped iterations. A random subset consisting of 20% of observations from each species and phenophase combination was held out from model fitting for later evaluation.

### Analysis

As described above, we fit two sets of models for each species and phenophase: one set of models parameterized using only USA‐NPN data, and one set parameterized using only LTER data (with the exception of the M1 and MSB models, see Models). We performed three primary analyses from these model outputs by comparing (1) the model parameters, (2) estimates from the models, and (3) out‐of‐sample errors from each model.

To compare the inferences about process made by the two data sets, we compared the distribution of each parameter between LTER and USA‐NPN derived models for each species and phenophase combination. Using the mean value of each bootstrapped parameter, we also calculated the coefficient of determination ($R^{2}$) between LTER and USA‐NPN derived models among the 38 species‐phenophases. In three cases where a species phenophase combination occurred in two LTER sites (Budburst for *Acer saccharum*,* Betula alleghaniensis*, and *Fagus grandifolia* in the Harvard and Hubbard Brook data sets), they were compared separately to the USA‐NPN data.

Next we compared the estimates of phenological events between models. Models with different parameter values, and even entirely different structures, can produce similar estimates for the date of phenological events (Basler 2016). Therefore, to compare the predictions and potential forecasts for models fit to the different data sets, we compared the estimated DOY predicted by the LTER and USA‐NPN derived models for all held out observations. For each of the eight models, we calculated the coefficient of determination ($R^{2}$) between LTER and USA‐NPN derived estimates for estimates made at the four LTER sites and across all USA‐NPN sites.

Finally, we directly evaluated performance using out‐of‐sample errors from the four combinations of models and observed data: (1) LTER‐derived models predicting LTER observations, (2) USA‐NPN derived models predicting LTER observations, (3) LTER‐derived models predicting USA‐NPN observations, and (4) USA‐NPN derived models predicting USA‐NPN observations. Using the RMSE values from held out observations, we compared the performance of LTER and USA‐NPN derived models on different data types in two different ways. First, we focused on local‐scale prediction by calculating the difference in the RMSE of LTER and USA‐NPN derived models solely with LTER observations. Second, to focus on large‐scale prediction, we calculated the difference in RMSE using solely USA‐NPN data. These differences were calculated for each of the model types and 38 species–phenophase combinations. Negative values indicate that LTER‐derived models perform better, while positive values indicate that the USA‐NPN derived model performed better. We used a *t* test to test the difference from zero in these values. In the three cases where the same species and phenophase combination occurred in two LTER sites, we made the LTER‐LTER comparison within each site, not across sites, to focus on local‐scale prediction when LTER data are available. Absolute RMSE values as well as Pearson correlation coefficients are provided in the supplement for specific species (Appendix S2: Figs. S5–S7) and with all observations aggregated together (Appendix S2: Table S2).

We performed all analysis using both the R and Python programming languages (R Core Team, 2017; Python Software Foundation, 2018). Primary R packages used in the analysis included dplyr (Wickham et al. 2017), tidyr (Wickham and Henry 2018), ggplot2 (Wickham 2016), lubridate (Grolemund and Wickham 2011), prism (Hart and Bell 2015), raster (Hijmans 2017), and sp (Pebesma and Bivand 2005). Primary Python packages included SciPy (Jones et al. 2001), NumPy (Oliphant 2006), Pandas (McKinney 2010), and MPI for Python (Dalcin et al. 2011). Code to fully reproduce this analysis is available online (see Data Availability).

## Results

Throughout the analysis there were no qualitative differences between a 30‐d or a 15‐d threshold between the first yes and most recent no observation in the USA‐NPN data set. Results presented here reflect the 30‐d cutoff; see the Figs. S2–S4 in Appendix S2 for matching figures using a 15‐d cutoff.

**Figure 2:**
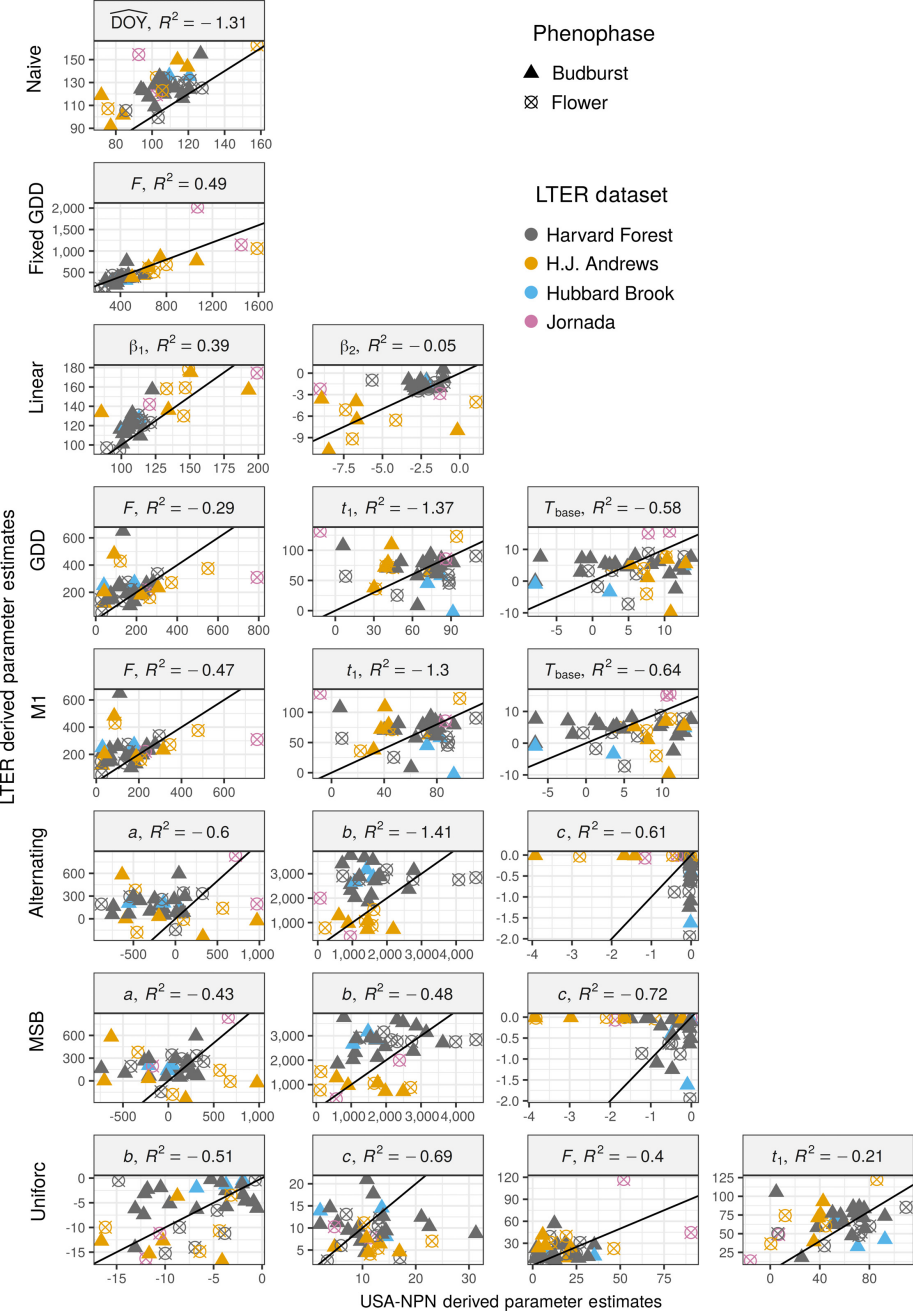
Comparisons of parameter estimates between USA National Phenology Network (USA‐NPN) and LTER derived models. Each point represents a parameter value for a specific species and phenophase and is the mean value from 250 bootstrap iterations. The black line is the 1:1 line. The $R^{2}$ is the coefficient of determination, which can be negative if the relationship between the two parameter sets is worse than no relationship but with the same mean values. Models and parameters are defined in Table 2.

The best matches between parameter estimates based on USA‐NPN and LTER data were the Fixed GDD model ($R^{2}$ = 0.49) and the Linear model ($R^{2}$ = 0.39 for $\beta_{1}$ and −0.05 for $\beta_{2}$). The parameters for all other models had $R^{2}$ values <0 indicating that the relationship was worse than no relationship between the parameters (but with matching mean parameter values across the two sets of models; Fig. 2). The Naive model showed a distinct late bias in mean DOY estimates for phenological events, likely resulting from the LTER data sets being mostly in the northern United States compared to the site locations of the USA‐NPN data set (Fig. 2). The large outlier for the Fixed GDD model is *Larrea tridentata*; this species’ flower phenology is largely driven by precipitation, which is not considered in the Fixed GDD model (Beatley 1974). While the Fixed GDD and Linear models showed reasonable correspondence between parameter estimates, all parameters for individual species and phenophase combinations had different distributions between USA‐NPN and LTER‐derived models (Appendix S2: Figs. S10 and S11).

**Figure 3:**
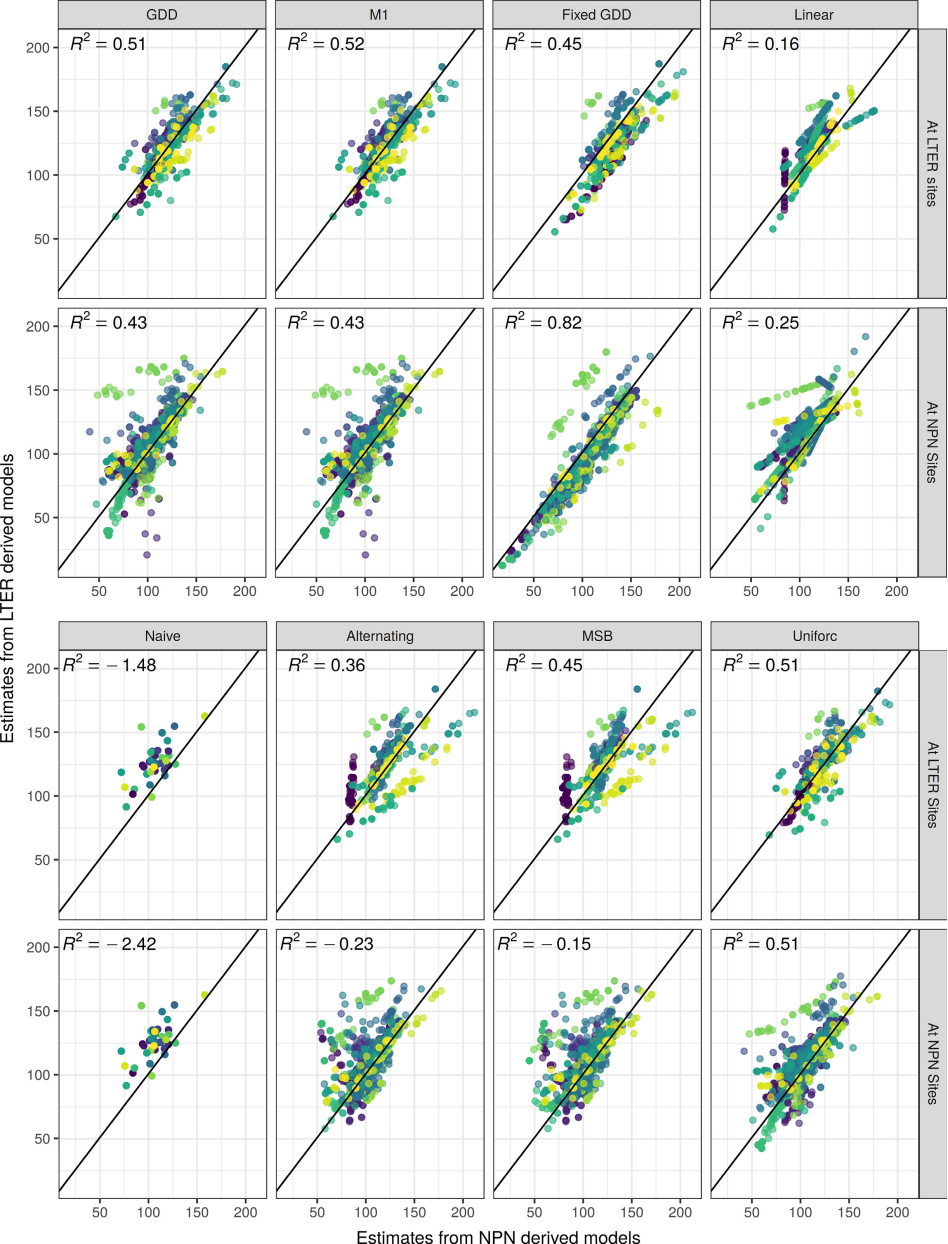
Comparison of predicted day of year (DOY) of all phenological events between USA‐NPN and LTER‐derived models. Top panels show comparisons at LTER sites and bottom panels show comparisons at USA‐NPN sites. Each point is an estimate for a single held‐out observation. Colors indicate observations for a single species and phenophase combination.

When comparing estimates of phenological events between the two sets of models, many USA‐NPN and LTER models produced similar estimates (Fig. 3). The Fixed GDD model had the highest correlation between the two model sets at USA‐NPN sites ($R^{2}=0.82$), while the GDD, M1, and Uniforc models had the highest correlation at LTER sites ($R^{2}$ = 0.51, 0.52, and 0.51, respectively). Comparing models with spatial corrections to the non‐spatial alternatives, the MSB (an extension of the Alternating model with a spatial correction based on mean spring temperature, see Table 2 and Methods) improved the correlation between the two data sets over the Alternating model. The MSB model improved the $R^{2}$ from 0.36 to 0.45 at LTER sites, and from −0.23 to −0.15 at USA‐NPN sites. The M1 model (an extension of the GDD model with a spatial correction based on day length) improved the correlation over the GDD model only slightly at LTER sites (from 0.51 to 0.52) and did not improve the correlation at USA‐NPN sites.

**Figure 4:**
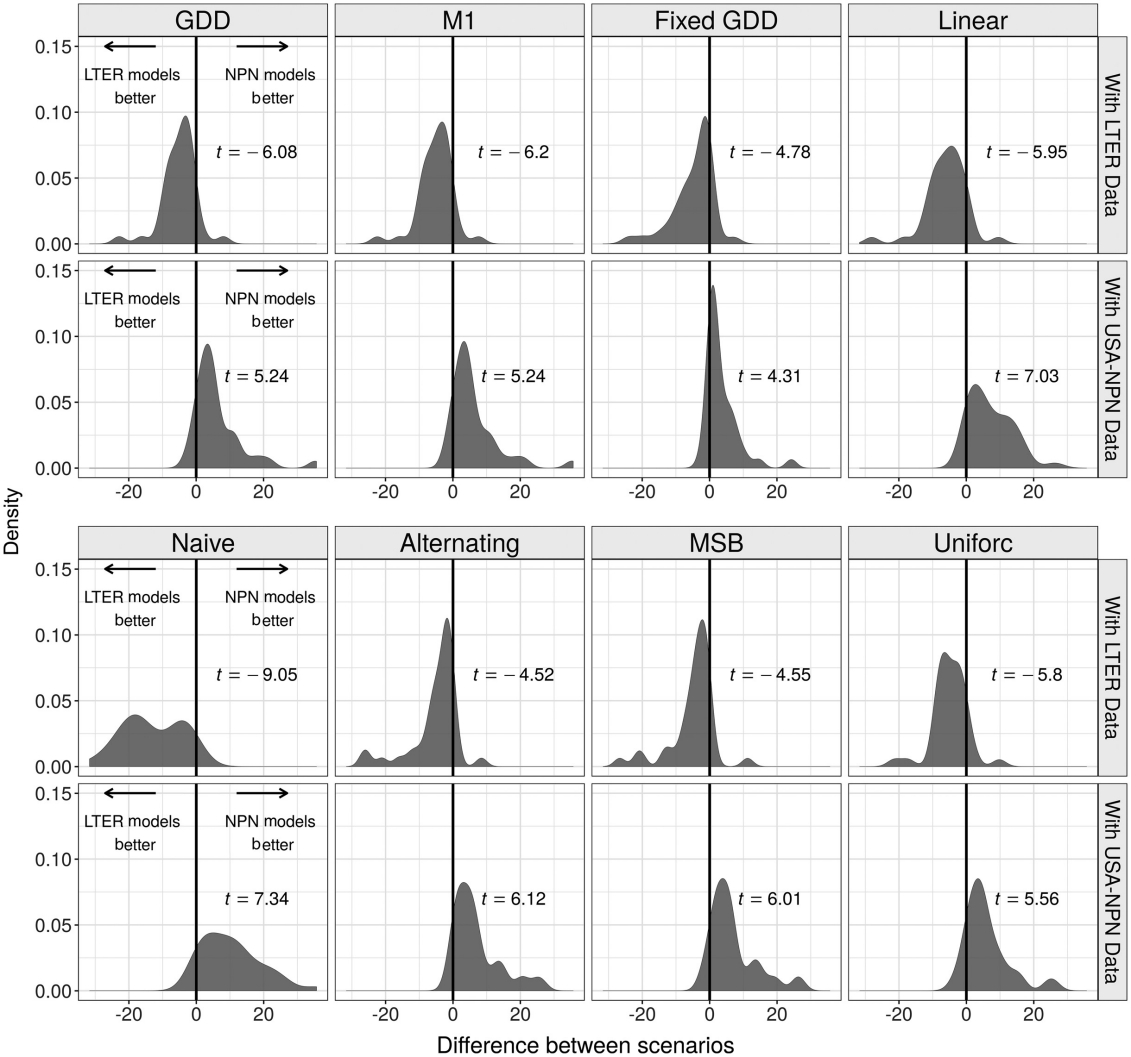
Differences in prediction error between USA‐NPN and LTER‐derived models. Density plots for comparisons of predictions on LTER data (top row) and USA‐NPN data (bottom row). Each plot represents the difference between the RMSE for LTER‐derived model and the USA‐NPN derived model, meaning that values less than zero indicate more accurate prediction by LTER‐derived models and values greater than zero indicate more accurate prediction by NPN‐derived models. *P *<* *0.001 for all *t* tests. Differences are calculated pairwise for the 38 species/phenophase comparisons.

When comparing the predictive performance using out‐of‐sample errors, USA‐NPN derived models made more accurate predictions for held‐out USA‐NPN observations, and LTER‐derived models performed better on held‐out LTER observations (all *P *<* *0.001; Fig. 4). The Naive and Linear models had the largest differences between the two model sets, while the Fixed GDD model had relatively similar errors when evaluated on both USA‐NPN and LTER held‐out observations. Although the Fixed GDD model had the highest agreement in accuracy between USA‐NPN and LTER‐derived models, it was not the best performing model overall. The GDD and Uniforc models made the best out of sample predictions, having the lowest RMSE and Pearson coefficient when aggregating all observations together (Appendix S2: Table S2). One exception was that the Fixed GDD model had a slightly higher Pearson value when using LTER‐derived models to make predictions for USA‐NPN data. The best model for specific species and phenophases varied, but was commonly the Uniforc or GDD models (Appendix S2: Figs. S5 and S6).

## Discussion

Data used to build phenology models typically fall into two categories: intensive long‐term data with long time‐series at a small number of locations (e.g., LTER data in this study), and large‐scale data with less intensive sampling at hundreds of locations (e.g., USA‐NPN data; Table 3). This data scenario, a small amount of intensive data and a large amount of less intensive data, is common in many areas of science and makes it necessary to understand how to choose between, or combine, data sources (Hanks et al. 2011). We explored this issue for phenology modeling in relation to making predictions and inferring process from models. For inference, we found that models based on different data sources resulted in different parameter estimates for all but the simplest models. For prediction we found that models fit to different data sources tended to make similar predictions, but that models better predicted out‐of‐sample data from the data type to which they were fit. These results are consistent with other research showing that phenology model performance decreases when transferring single‐site models to other locations (García‐Mozo et al. 2008, Xu and Chen 2013, Basler 2016), and with the call for models that better incorporate spatial variation in phenology requirements (Richardson et al. 2013, Chuine and Régnière 2017). Understanding and making predictions for the phenology of a single location is best served by intensive local‐scale data, when available, but large‐scale data sets work better for extrapolating phenology predictions across a species range. Thus, the best choice of both data and models depends on the desired research goals.

**Table 3:** Attributes of the two data sets used in this study

Parameter	LTER	USA‐NPN
Time‐series length	**High**	**Low**
Spatial extent	Low	**High**
Local species representation	High	Low
Regional/Continental species representation	Low	**High**
Number of observers	**Low**	**High**
Site fidelity	High	Low

*Note*

Bold text indicates an attribute is expected to increase over time.

In this study, parameter estimates differed widely within the same phenology model when fit to the two different types of data, except for the simplest process‐oriented model: the Fixed GDD (Fig. 2). These differences may be caused by a variety of factors that have different implications for interpreting process‐oriented models and their parameters. First, the differences could result from limitations in the sampling of the USA‐NPN data set, such as irregular sampling of the same location within or between seasons, leading to less accurate parameter estimates. If this is the case, it would suggest that using LTER data is ideal for making inferences about plant physiology, and that focusing on the Fixed GDD model is best for making inferences when USA‐NPN data are all that is available. Second, spatial variation (e.g., from local adaptation, acclimation, microclimates, or plant age) in phenology requirements and drivers could contribute to these differences (Diez et al. 2012, Zhang et al. 2017). Models built using USA‐NPN data integrate over that spatial variation, while models built using LTER data only estimate the phenological requirements for a specific site. In this case, USA‐NPN data would provide a better estimate of the general phenological requirements of a species, but LTER data would provide a more accurate understanding for a single site. The best solution to this issue would be the development of models that accurately incorporate spatial variation, such as including genetic variation between different populations (Chuine and Régnière 2017), although localized models could also be generated when large‐scale predictions are unnecessary. Third, these differences could result from issues with model identifiability: since different parameter values can yield nearly identical estimates of phenological events, parameter estimates can differ between data sets even when the underlying processes generating the data are the same. Information about which of these issues may be causing the differences between data sets can be explored using the analyses in the current study, as will be explained below.

Despite substantial differences in parameter estimates, LTER and USA‐NPN derived models produced similar estimates for phenological events in most cases (Fig. 3). This greater correspondence between predictions than parameters suggests that more complex models may have identifiability issues. For example, two GDD models with parameters of $t_{1}$ = 1, F = 10, $T_{\mathrm{base}}$ = 0 and $t_{1}$ = 5, F = 5, $T_{\mathrm{base}}$ = 0 produce nearly identical estimates in many scenarios. This possibility is supported by the fact that the highest correlation between parameter estimates is seen in models with only one or two parameters. In addition, bootstrap results for more complex models suggest a high degree of variability in parameter estimates and potentially multiple local optima in fits to both USA‐NPN and LTER data (Appendix S2: Figs. S10 and S11). Finally, parameter estimates of more complex models are also not consistent among models for the same species when comparing multiple LTER data sets (Appendix S2: Figs. S8 and S9). These results are consistent with research showing that models failed to estimate the starting day of warming accumulation solely from budbreak time‐series, thus producing parameter estimates that were not biologically realistic (Chuine et al. 2016). Basler (2016) suggests that the key component in phenology models is the thermal forcing, with additional parameters being sensitive to over‐fitting. Here, our simplest model, the Fixed GDD model, which uses only a warming component, had the highest correlation among parameters between LTER and USA‐NPN data sets. In combination with the aforementioned studies, our results indicate that caution is warranted in interpreting parameter estimates from complex phenology models regardless of the data source used for fitting the models.

While more complex phenology models appear to have identifiability issues, there is also evidence that they capture useful information, beyond the Fixed GDD model, based on their ability to make out‐of‐sample predictions. Based on the RMSE, the GDD and Uniforc models produce the best out‐of‐sample predictions for the majority of species and phenophases at both USA‐NPN and LTER data sets (Appendix S2: Figs. S5 and S6). This demonstrates that the more complex models are capturing additional information about phenology, and that some of the differences between data sets result from differences in either the scales or the sampling of the data. Spatial variation in phenological requirements is known to exist in plants (Zhang et al. 2017). In combination with our results showing observed differences in parameter estimates between LTER sites (Appendix S2: Figs. S8 and S9), this suggests that variation in phenological requirements across the range is likely important. However, the models that attempted to address this by incorporating spatial variation did not yield improvements over their base models in our analyses. Specifically, correspondence between parameter estimates (Fig. 2), estimates of phenological events (Fig. 3), and out‐of‐sample error rates (Fig. 4) for the MSB and M1 models were essentially the same as the Alternating and GDD models, respectively. This lack of improvement from incorporating spatial variation could be caused either by models not adequately capturing the process driving the spatial variation, the USA‐NPN data set having biases from variation in sampling effort and/or spatial auto‐correlation, or some combination of these factors. Basler (2016) used the M1 model to predict budburst for six species across Europe and found it was generally among the best models in terms of RMSE, albeit never by more than a single day. Their result was strengthened by having a 40‐yr time‐series across a large region. Chuine and Régnière (2017) listed the incorporation of spatial variation in warming requirements in models as a primary issue in future phenology research. Large‐scale phenology data sets, like USA‐NPN, will be key in addressing this and other phenological research needs.

In addition to exploring differences between phenology data sets, our analyses provide guidance on which models to use when making predictions at a local scale using models built from large‐scale data, or vice versa. Among the eight models tested, the Uniforc and GDD models performed the best overall in the cross data set comparison in terms of Pearson correlation and RMSE (Appendix S2: Figs. S5 and S6, Table S2). The GDD model has one less parameter than the Uniforc model, thus the GDD model is a suitable choice for making predictions when there is little to no information at the location of interest (e.g., making phenology forecasts at a new location distant from any observed data). This guidance can vary between species, though, and model testing should still be performed when suitable data are available.

In conclusion, our results suggest that both LTER and USA‐NPN data provide valuable information on plant phenology. Models built using both data sources yield effective predictions for phenological events, but parameter estimates from the two data sources differ and models from each source best predict that data source's phenology events. The primary difference in the data sets is spatial scale, but due to trade‐offs in data collection efforts, the larger scale USA‐NPN data have shorter time series, less site fidelity and other differences from the intensively collected LTER data (Table 3). These differences can be strengths or potential limitations. Observers sampling opportunistically allows the USA‐NPN data set to have a large spatial scale, but also leads to low site fidelity, which limits the ability to measure long‐term trends at local scales (Gerst et al. 2016). Tracking long‐term trends is the major strength of LTER data, but having a relatively small species pool limits their use in species‐level predictive modeling. Due to these differences, the best data source for making predictions depends on the scale at which the predictions are being made. Identifying the most effective data sources for different types and scales of analysis is a useful first step, but the ultimate solution to working with diverse data types is to focus on integrating all types of data into analyses and forecasts (Hanks et al. 2011, Melaas et al. 2016). Our results suggest that methods that can learn from the intensive information available in LTER data in regions where they are available, and simultaneously use large‐scale data to capture spatial variation in phenological requirements will help improve our ability to understand and predict phenology. Data integration efforts should also leverage data from remote sensing sources such as the PHENOCAM network or satellite imagery, which have both a large spatial extent and high temporal resolution (Peng et al. 2017, Richardson et al. 2018a *,* b). Data integration provides the potential to use data from many sources to produce the best opportunity for accurate inference about, and forecasting of, the timing of biological events.

## Data Availability

Code to fully reproduce this analysis is available on GitHub at https://github.com/sdtaylor/phenology_dataset_study and archived on Zenodo at https://doi.org/10.5281/zenodo.1256705.
