# Supplementary material for: Comparison of large‐scale citizen science data and long‐term study data for phenology modeling
Source: Ecology. 2018 Dec 24;100(2):e02568. doi: 10.1002/ecy.2568 (PMC7378950; doi:10.1002/ecy.2568)
Supplement: Supplementary file 1 [file ECY-100-e02568-s001.pdf]

Appendix S1
AppendixS1.pdf

Comparison of large-scale citizen science data and long-term study data for phenology modeling

Shawn D. Taylor, Joan M. Meiners, Kristina Riemer, Michael C. Orr, Ethan P. White

Supplementary materials

Methods describing processing of the 4 LTER datasets

The four LTER datasets each had different protocols for recording phenology observations. Below are details for converting the data from each to a status based yes/no as used in the National Phenology Network. As in the USA-NPN datasets, the julian day of year (DOY) used in modeling was the midpoint between each "yes" observation and the most recent "no" observation. The years used for each datasets were all years available at the time of analysis.

Harvard Forest

We used observations from 1990-2014. Observations here were recorded as relative percentage of flowering or budburst for individual plants. We set "Yes" observations budburst and flower to the DOY when the percentage of each tree had was greater than or equal to 10%. Harvard Forest has a sampling interval of 3-7 days.

H.J. Andrews Experimental Forest

We used observations from 2009-2015. We set "yes" observations for budburst to the first DOY when an individual was marked as "bud break" and "yes" observations for flowering were when an individual was first marked as "Flowers open". Note that each species has slightly different ordinal codes to mark each of these events. H.J. Andrews has a sampling interval of 7 days.

Jornada Experimental Range

We used observations from 1992-2009. Observations for this dataset represent, for each zone, the percent of plants for a particular species which is observed within each phenophase. We set "Yes" observations for flowers to the first DOY where the flower phenophase was 10% or greater. Jornada has a sampling interval of 30 days.

Hubbard Brook

We used observations from 1989-2015. Observations for this dataset represent for each species the average, among 3 individuals, of an ordinal description of phenophase.
0: winter conditions
1: bud swelling
2: small leaves or flowers
3: leafs 1/2 of final length, leafs obscure half the sky as seen thru crown
3.5: leaves 3/4 expanded, sky mostly obscured, crown not yet in summer condition
4: fully expanded, canopy in summer conditions

We set "Yes" observations for budburst to the DOY when the average value was greater than or equal to 1.6. This is the value where the 3 individuals most likely have ordinal observations of [1,2,2]. Hubbard Brook has a sampling interval of 7 days.
