# Supplementary material for: Comparison of large‐scale citizen science data and long‐term study data for phenology modeling
Source: Ecology. 2018 Dec 24;100(2):e02568. doi: 10.1002/ecy.2568 (PMC7378950; doi:10.1002/ecy.2568)
Supplement: Supplementary file 2 [file ECY-100-e02568-s002.pdf]

Appendix S2

AppendixS2.pdf

Comparison of large-scale citizen science data and long-term study data for phenology modeling

Shawn D. Taylor, Joan M. Meiners, Kristina Riemer, Michael C. Orr, Ethan P. White

Supplementary materials

Supplementary images S1 - S11 and Tables S1-S2

Figure S1: Sensitivity test results from using a 15 versus 30 day cutoff between the 'yes' and most recent 'no' in the USA-NPN dataset. Each point represents the value of a single parameter for one of eight models for 32 unique species/phenophase combinations. This is from 23 species with varying combinations of the budburst and flowering phenophases (see Table S1). Only 32 comparisons were possible here as the stricter 15 day cutoff resulted in three species/phenophase combinations not having sufficient observations. This figure does not include any data from the LTER datasets. Note that the primary analysis states 35 combinations using the 15 day cutoff. This is because 3 species are duplicated in the Harvard Forest and Hubbard Brook datasets, thus 3 extra comparisons are available for the primary analysis.

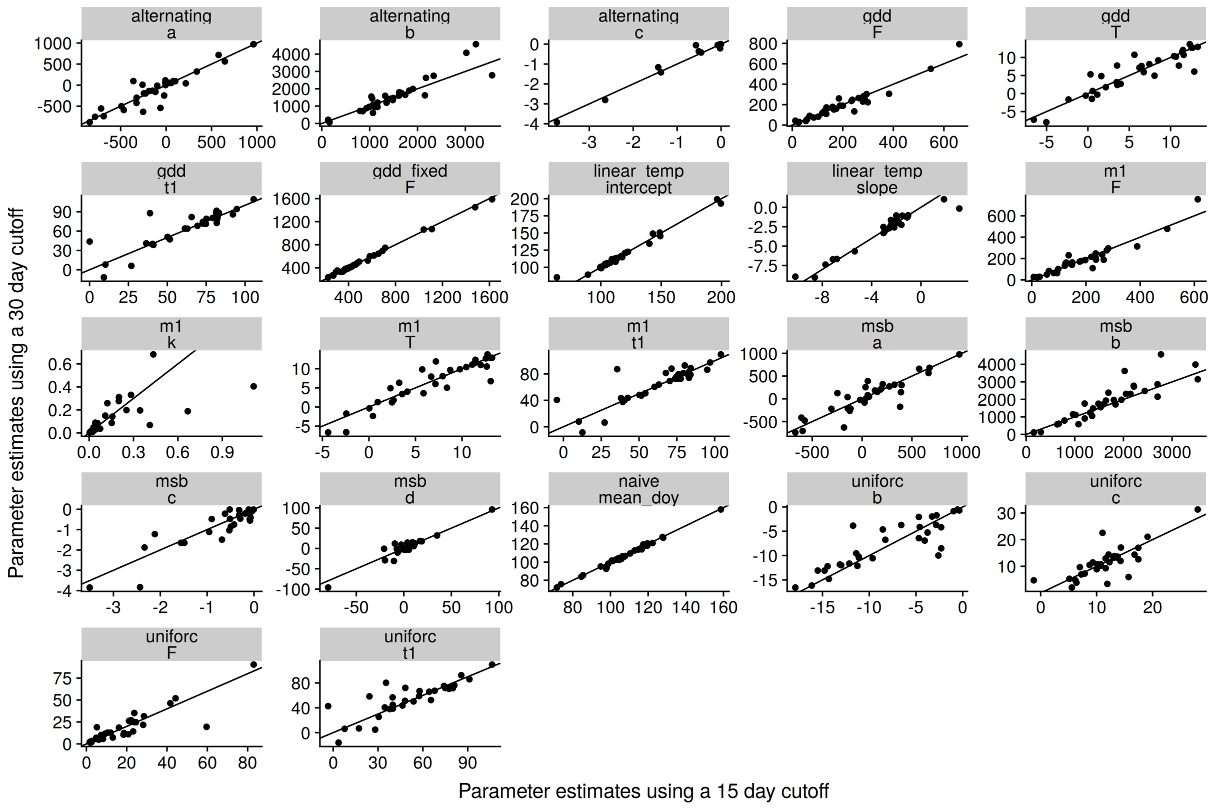
alternating a
alternating b
alternating c
gdd F
gdd T
gdd t1
gdd_fixed F
linear_temp intercept
linear_temp slope
m1 F
m1 k
m1 T
m1 t1
msb a
msb b
msb c
msb d
naive mean_doy
uniforc b
uniforc c
uniforc F
uniforc t1
Parameter estimates using a 30 day cutoff
Parameter estimates using a 15 day cutoff

Figure S1

Figure S2: Comparisons of parameter estimates between NPN and LTER derived models. As in Figure 2 in the main text, but using a threshold of 15 instead of 30 days between the first 'yes' and most recent 'no' in the USA-NPN dataset. See methods for details.

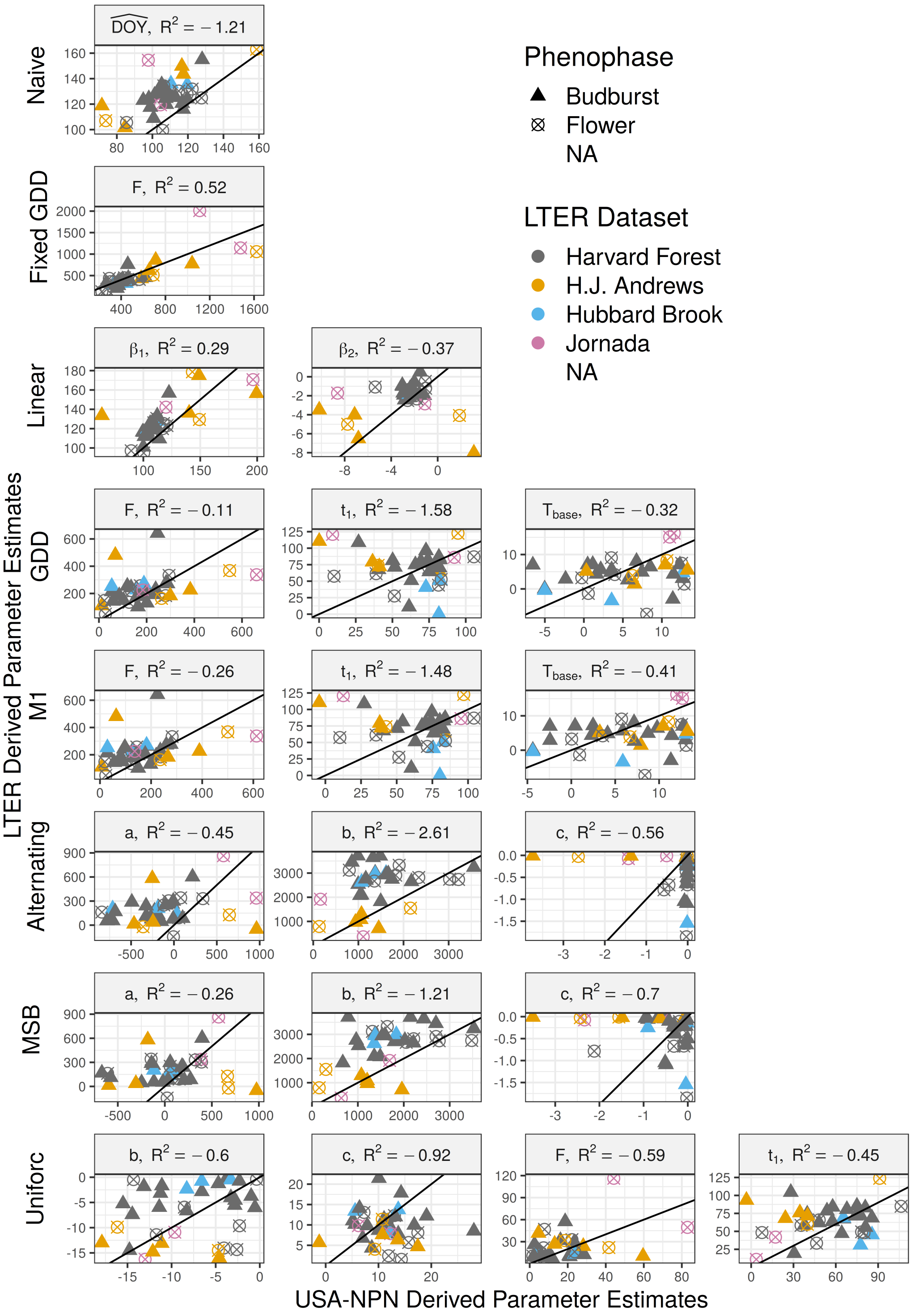
Phenophase
Budburst
Flower
NA
LTER Dataset
Harvard Forest
H.J. Andrews
Hubbard Brook
Jornada
NA
Naive
Fixed GDD
Linear
GDD
M1
Alternating
MSB
Uniforc
LTER Derived Parameter Estimates
USA-NPN Derived Parameter Estimates
$\widehat{DOY}$, $R^2 = -1.21$
F, $R^2 = 0.52$
β_1, $R^2 = 0.29$
β_2, $R^2 = -0.37$
F, $R^2 = -0.11$
t_1, $R^2 = -1.58$
T_{base}, $R^2 = -0.32$
F, $R^2 = -0.26$
t_1, $R^2 = -1.48$
T_{base}, $R^2 = -0.41$
a, $R^2 = -0.45$
b, $R^2 = -2.61$
c, $R^2 = -0.56$
a, $R^2 = -0.26$
b, $R^2 = -1.21$
c, $R^2 = -0.7$
b, $R^2 = -0.6$
c, $R^2 = -0.92$
F, $R^2 = -0.59$
t_1, $R^2 = -0.45$

Figure S2

Figure S3: Comparison of predicted day of year (DOY) of all phenological events between NPN and LTER-derived models. As in Figure 3 in the main text, but using a threshold of 15 instead of 30 days between the first 'yes' and most recent 'no' in the USA-NPN dataset. See methods for details.

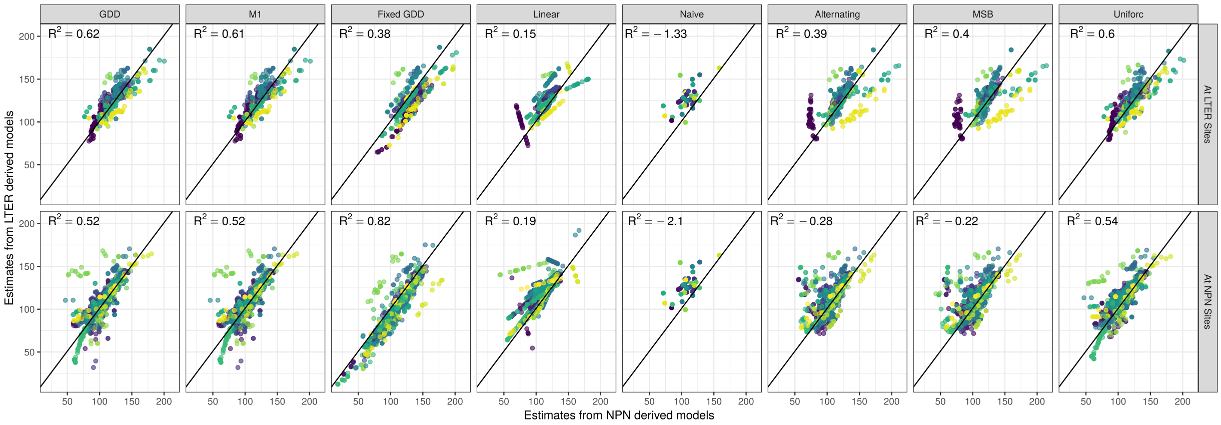
GDD
M1
Fixed GDD
Linear
Naive
Alternating
MSB
Uniforc
$R^2 = 0.62$
$R^2 = 0.61$
$R^2 = 0.38$
$R^2 = 0.15$
$R^2 = -1.33$
$R^2 = 0.39$
$R^2 = 0.4$
$R^2 = 0.6$
$R^2 = 0.52$
$R^2 = 0.52$
$R^2 = 0.82$
$R^2 = 0.19$
$R^2 = -2.1$
$R^2 = -0.28$
$R^2 = -0.22$
$R^2 = 0.54$
At LTER Sites
At NPN Sites
Estimates from LTER derived models
Estimates from NPN derived models

Figure S3

Figure S4: Differences in prediction error between NPN and LTER-derived models. As in Figure 4 in the main text, but using a threshold of 15 instead of 30 days between the first 'yes' and most recent 'no' in the USA-NPN dataset. See methods for details.

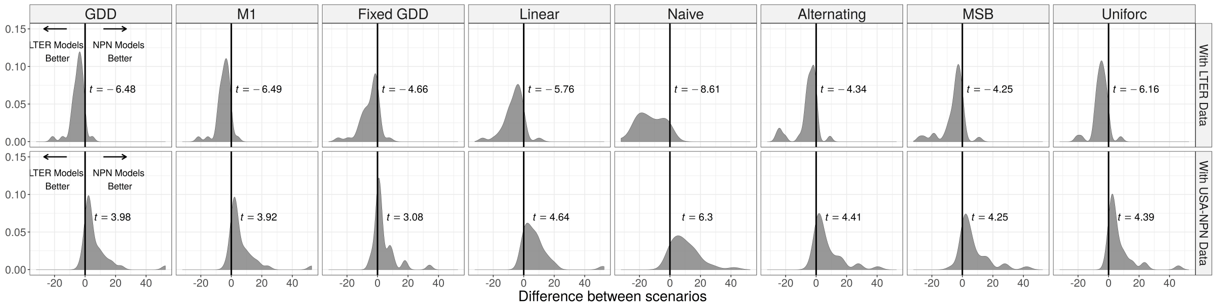
GDD
M1
Fixed GDD
Linear
Naive
Alternating
MSB
Uniforc
With LTER Data
With USA-NPN Data
LTER Models Better
NPN Models Better
t = −6.48
t = −6.49
t = −4.66
t = −5.76
t = −8.61
t = −4.34
t = −4.25
t = −6.16
t = 3.98
t = 3.92
t = 3.08
t = 4.64
t = 6.3
t = 4.41
t = 4.25
t = 4.39
Difference between scenarios

Figure S4

Figure S5: RMSE for specific species and phenophases using all combinations of models and data sources. Red X's mark the best performing models for each respective dataset.

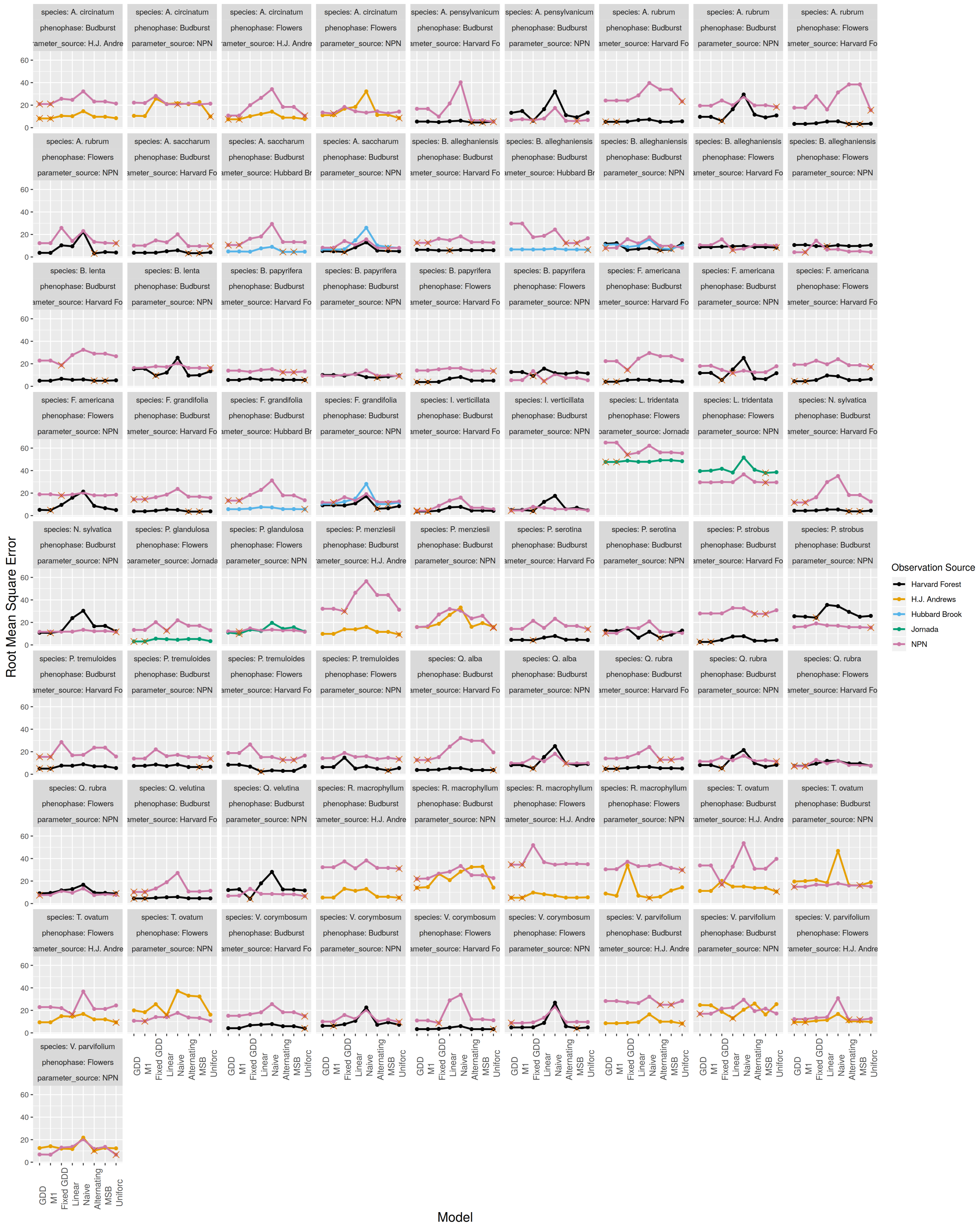

Figure S5

Figure S6: Pearson correlation coefficients for specific species and phenophases using all combinations of models and data sources. Note that since all predictions from each Naive model are the same the Pearsons correlation cannot be calculated here. Red X's mark the best performing models for each respective dataset.

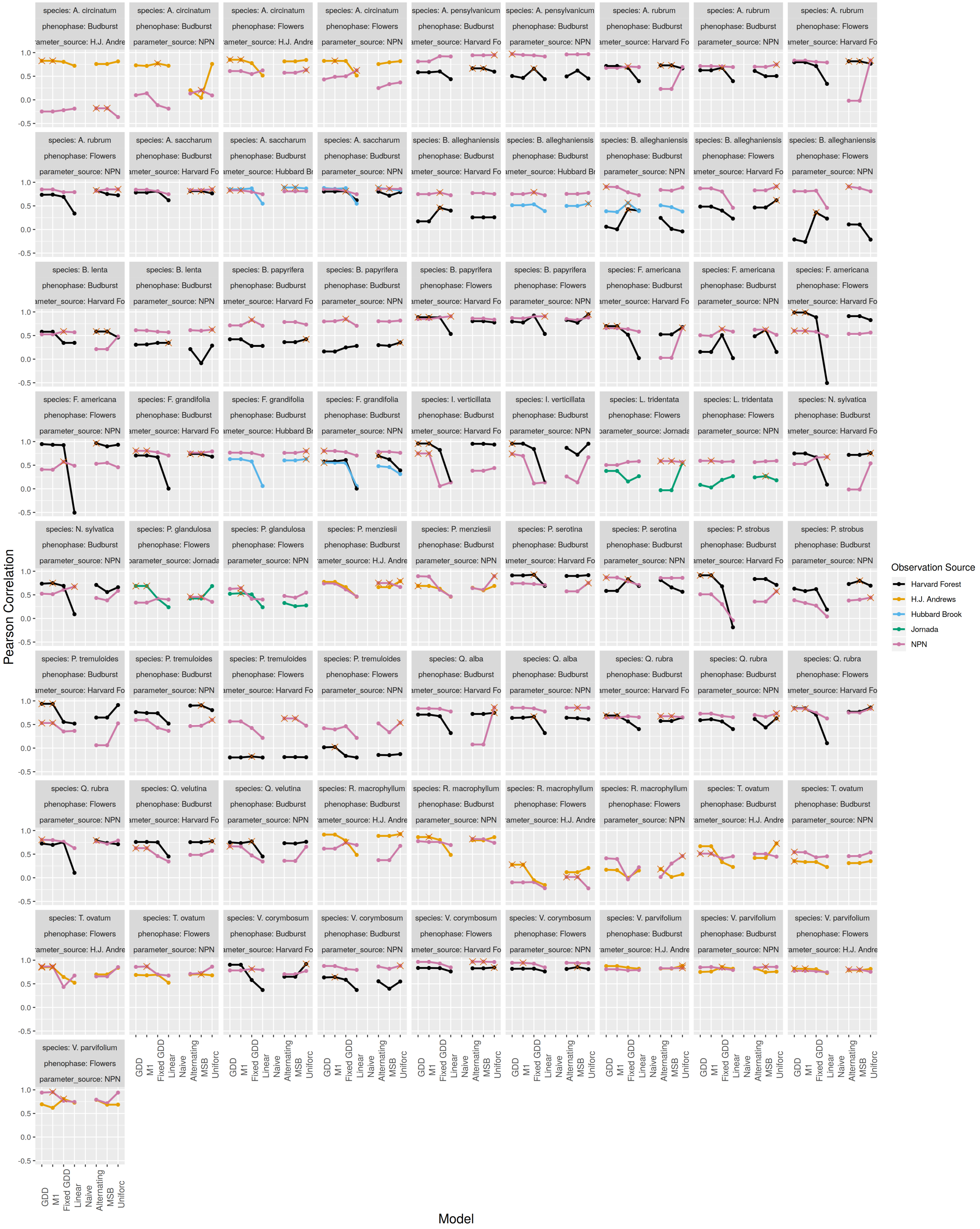
species: A. circinatum
phenophase: Budburst
rameter_source: H.J. Andre
species: A. circinatum
phenophase: Budburst
parameter_source: NPN
species: A. circinatum
phenophase: Flowers
rameter_source: H.J. Andre
species: A. circinatum
phenophase: Flowers
parameter_source: NPN
species: A. pensylvanicum
phenophase: Budburst
ameter_source: Harvard Fo
species: A. pensylvanicum
phenophase: Budburst
parameter_source: NPN
species: A. rubrum
phenophase: Budburst
ameter_source: Harvard Fo
species: A. rubrum
phenophase: Budburst
parameter_source: NPN
species: A. rubrum
phenophase: Flowers
ameter_source: Harvard Fo
species: A. rubrum
phenophase: Flowers
parameter_source: NPN
species: A. saccharum
phenophase: Budburst
ameter_source: Harvard Fo
species: A. saccharum
phenophase: Budburst
ameter_source: Hubbard Br
species: A. saccharum
phenophase: Budburst
parameter_source: NPN
species: B. alleghaniensis
phenophase: Budburst
ameter_source: Harvard Fo
species: B. alleghaniensis
phenophase: Budburst
ameter_source: Hubbard Br
species: B. alleghaniensis
phenophase: Budburst
parameter_source: NPN
species: B. alleghaniensis
phenophase: Flowers
ameter_source: Harvard Fo
species: B. alleghaniensis
phenophase: Flowers
parameter_source: NPN
species: B. lenta
phenophase: Budburst
ameter_source: Harvard Fo
species: B. lenta
phenophase: Budburst
parameter_source: NPN
species: B. papyrifera
phenophase: Budburst
ameter_source: Harvard Fo
species: B. papyrifera
phenophase: Budburst
parameter_source: NPN
species: B. papyrifera
phenophase: Flowers
ameter_source: Harvard Fo
species: B. papyrifera
phenophase: Flowers
parameter_source: NPN
species: F. americana
phenophase: Budburst
ameter_source: Harvard Fo
species: F. americana
phenophase: Budburst
parameter_source: NPN
species: F. americana
phenophase: Flowers
ameter_source: Harvard Fo
species: F. americana
phenophase: Flowers
parameter_source: NPN
species: F. grandifolia
phenophase: Budburst
ameter_source: Harvard Fo
species: F. grandifolia
phenophase: Budburst
ameter_source: Hubbard Br
species: F. grandifolia
phenophase: Budburst
parameter_source: NPN
species: I. verticillata
phenophase: Budburst
ameter_source: Harvard Fo
species: I. verticillata
phenophase: Budburst
parameter_source: NPN
species: L. tridentata
phenophase: Flowers
parameter_source: Jornada
species: L. tridentata
phenophase: Flowers
parameter_source: NPN
species: N. sylvatica
phenophase: Budburst
ameter_source: Harvard Fo
species: N. sylvatica
phenophase: Budburst
parameter_source: NPN
species: P. glandulosa
phenophase: Flowers
parameter_source: Jornada
species: P. glandulosa
phenophase: Flowers
parameter_source: NPN
species: P. menziesii
phenophase: Budburst
ameter_source: H.J. Andre
species: P. menziesii
phenophase: Budburst
parameter_source: NPN
species: P. serotina
phenophase: Budburst
ameter_source: Harvard Fo
species: P. serotina
phenophase: Budburst
parameter_source: NPN
species: P. strobus
phenophase: Budburst
ameter_source: Harvard Fo
species: P. strobus
phenophase: Budburst
parameter_source: NPN
species: P. tremuloides
phenophase: Budburst
ameter_source: Harvard Fo
species: P. tremuloides
phenophase: Budburst
parameter_source: NPN
species: P. tremuloides
phenophase: Flowers
ameter_source: Harvard Fo
species: P. tremuloides
phenophase: Flowers
parameter_source: NPN
species: Q. alba
phenophase: Budburst
ameter_source: Harvard Fo
species: Q. alba
phenophase: Budburst
parameter_source: NPN
species: Q. rubra
phenophase: Budburst
ameter_source: Harvard Fo
species: Q. rubra
phenophase: Budburst
parameter_source: NPN
species: Q. rubra
phenophase: Flowers
ameter_source: Harvard Fo
species: Q. rubra
phenophase: Flowers
parameter_source: NPN
species: Q. velutina
phenophase: Budburst
ameter_source: Harvard Fo
species: Q. velutina
phenophase: Budburst
parameter_source: NPN
species: R. macrophyllum
phenophase: Budburst
ameter_source: H.J. Andre
species: R. macrophyllum
phenophase: Budburst
parameter_source: NPN
species: R. macrophyllum
phenophase: Flowers
ameter_source: H.J. Andre
species: R. macrophyllum
phenophase: Flowers
parameter_source: NPN
species: T. ovatum
phenophase: Budburst
ameter_source: H.J. Andre
species: T. ovatum
phenophase: Budburst
parameter_source: NPN
species: T. ovatum
phenophase: Flowers
ameter_source: H.J. Andre
species: T. ovatum
phenophase: Flowers
parameter_source: NPN
species: V. corymbosum
phenophase: Budburst
ameter_source: Harvard Fo
species: V. corymbosum
phenophase: Budburst
parameter_source: NPN
species: V. corymbosum
phenophase: Flowers
ameter_source: Harvard Fo
species: V. corymbosum
phenophase: Flowers
parameter_source: NPN
species: V. parvifolium
phenophase: Budburst
ameter_source: H.J. Andre
species: V. parvifolium
phenophase: Budburst
parameter_source: NPN
species: V. parvifolium
phenophase: Flowers
ameter_source: H.J. Andre
species: V. parvifolium
phenophase: Flowers
parameter_source: NPN
Pearson Correlation
1.0
0.5
0.0
-0.5
GDD
M1
Fixed GDD
Linear
Naive
Alternating
MSB
Uniforc
Model
Observation Source
Harvard Forest
H.J. Andrews
Hubbard Brook
Jornada
NPN

Figure S6

Figure S7: RMSE of all species and phenophases of the four scenarios described in the text. These values were calculated using held out test data.

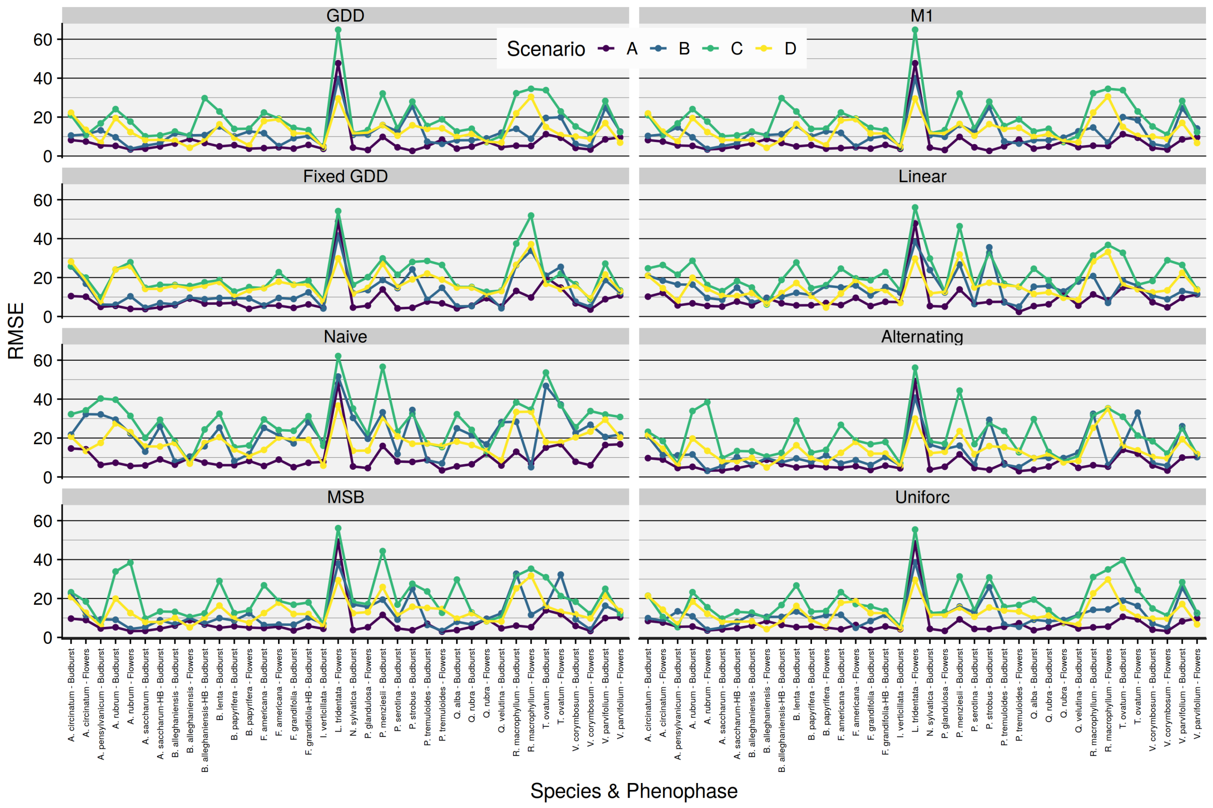
GDD
M1
Scenario A B C D
Fixed GDD
Linear
Naive
Alternating
MSB
Uniforc
RMSE
0
20
40
60
A. circinatum - Budburst
A. circinatum - Flowers
A. pensylvanicum - Budburst
A. rubrum - Budburst
A. rubrum - Flowers
A. saccharum - Budburst
A. saccharum-HB - Budburst
B. alleghaniensis - Budburst
B. alleghaniensis - Flowers
B. alleghaniensis-HB - Budburst
B. lenta - Budburst
B. papyrifera - Budburst
B. papyrifera - Flowers
F. americana - Budburst
F. americana - Flowers
F. grandifolia - Budburst
F. grandifolia-HB - Budburst
I. verticillata - Budburst
L. tridentata - Flowers
N. sylvatica - Budburst
P. glandulosa - Flowers
P. menziesii - Budburst
P. serotina - Budburst
P. strobus - Budburst
P. tremuloides - Budburst
P. tremuloides - Flowers
Q. alba - Budburst
Q. rubra - Budburst
Q. rubra - Flowers
Q. velutina - Budburst
R. macrophyllum - Budburst
R. macrophyllum - Flowers
T. ovatum - Budburst
T. ovatum - Flowers
V. corymbosum - Budburst
V. corymbosum - Flowers
V. parvifolium - Budburst
V. parvifolium - Flowers
Species & Phenophase

Figure S7

Figure S8: Distribution of parameters of the Naive, GDD, Fixed GDD, and Linear models for the three species common to the Hubbard Brook, Harvard, and USA-NPN datasets. The phenophase is budburst for all three species. Vertical lines indicate either the mean (solid) or median (dashed) of the respective distribution. Note the heading for each sub figure.

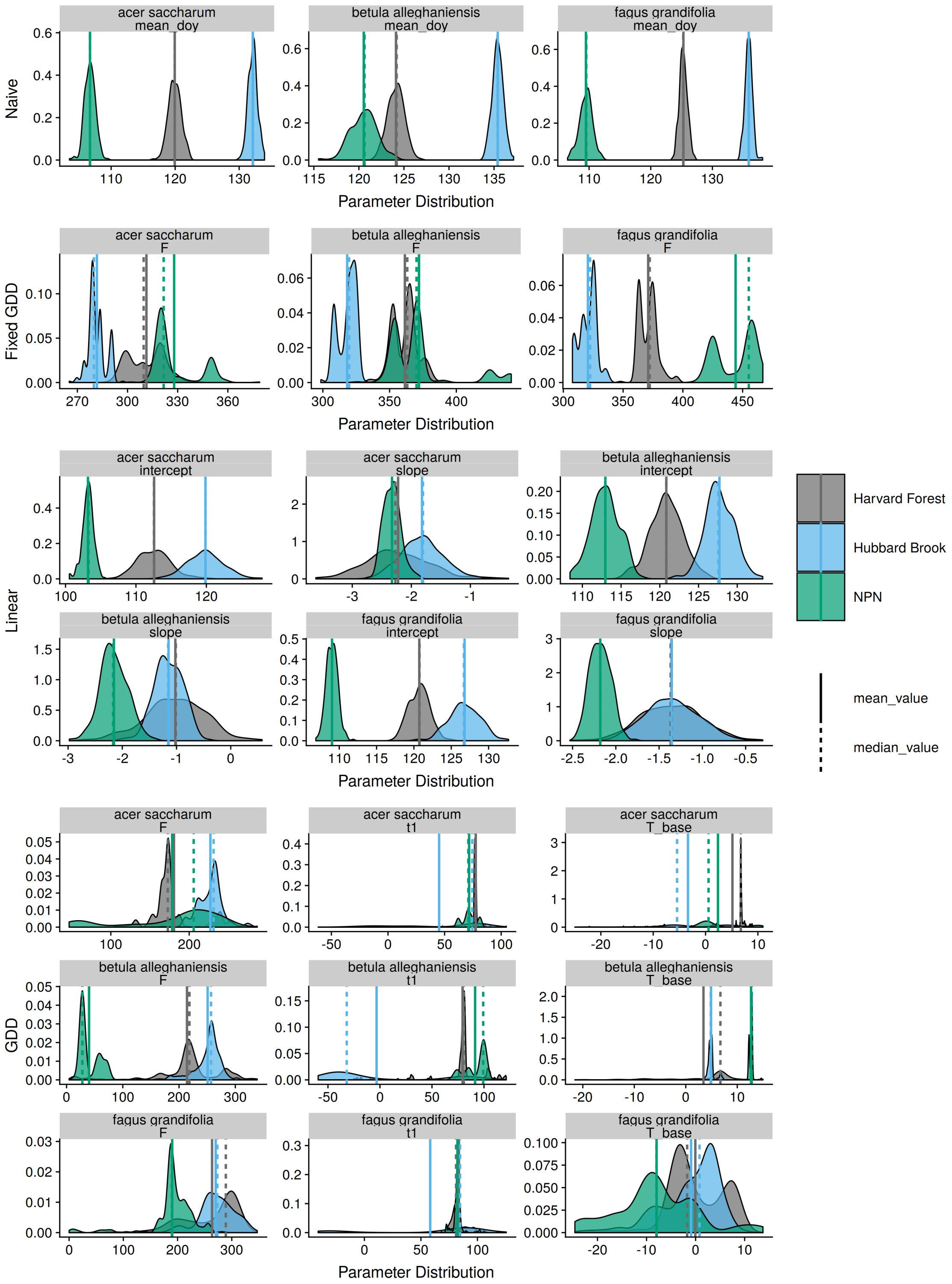

acer saccharum mean_doy
betula alleghaniensis mean_doy
fagus grandifolia mean_doy
Naive
Parameter Distribution
acer saccharum F
betula alleghaniensis F
fagus grandifolia F
Fixed GDD
Parameter Distribution
acer saccharum intercept
acer saccharum slope
betula alleghaniensis intercept
betula alleghaniensis slope
fagus grandifolia intercept
fagus grandifolia slope
Linear
Parameter Distribution
Harvard Forest
Hubbard Brook
NPN
mean_value
median_value
acer saccharum F
acer saccharum t1
acer saccharum T_base
betula alleghaniensis F
betula alleghaniensis t1
betula alleghaniensis T_base
fagus grandifolia F
fagus grandifolia t1
fagus grandifolia T_base
GDD
Parameter Distribution

Figure S8

Figure S9: As in Figure S4, but for the Alternating and Uniforc models.

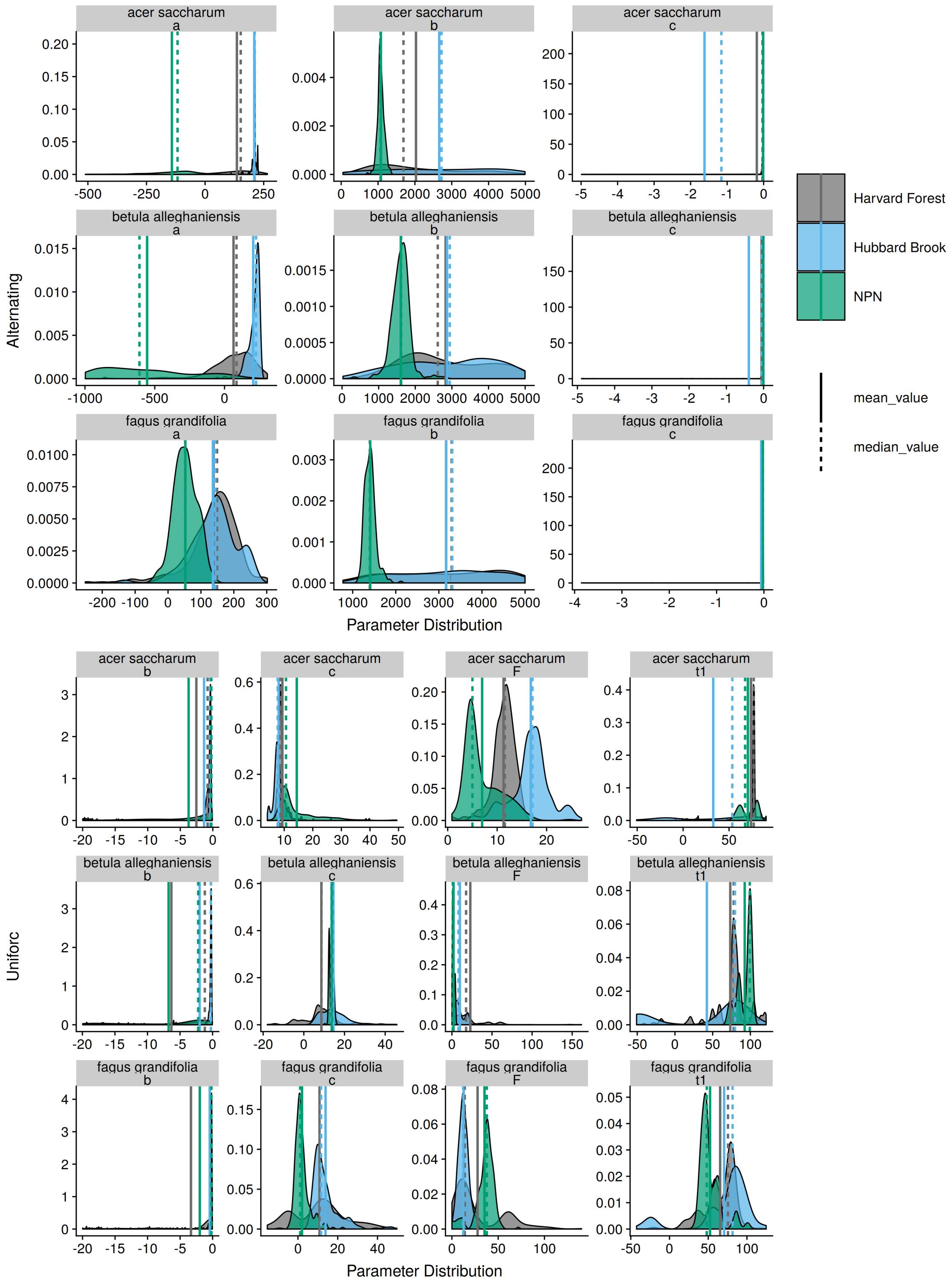

acer saccharum a
acer saccharum b
acer saccharum c
betula alleghaniensis a
betula alleghaniensis b
betula alleghaniensis c
fagus grandifolia a
fagus grandifolia b
fagus grandifolia c
Alternating
Parameter Distribution
Harvard Forest
Hubbard Brook
NPN
mean_value
median_value
acer saccharum b
acer saccharum c
acer saccharum F
acer saccharum t1
betula alleghaniensis b
betula alleghaniensis c
betula alleghaniensis F
betula alleghaniensis t1
fagus grandifolia b
fagus grandifolia c
fagus grandifolia F
fagus grandifolia t1
Uniforc
Parameter Distribution

Figure S9

Figure S10: As in Figure S4, but for 4 selected species to show the difference in parameter distributions between LTER and USA-NPN derived models. The phenophase for the four species is budburst. These 4 species are representative of the analysis, and for the remaining comparisons the reader is pointed to the script 'analysis/plot_select_species_parameters.R' in the code repository to generate additional figures.

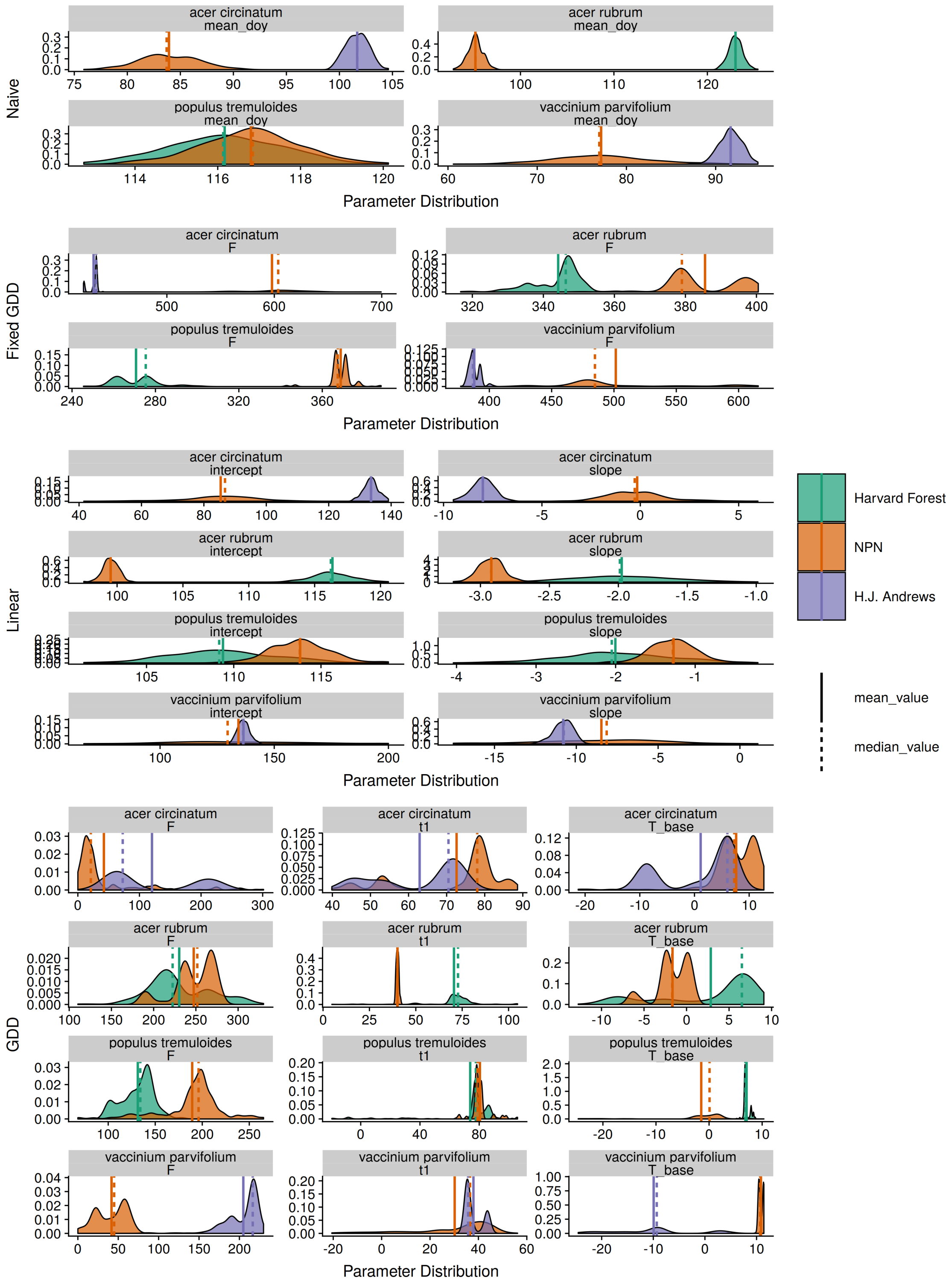
Naive
acer circinatum mean_doy
acer rubrum mean_doy
populus tremuloides mean_doy
vaccinium parvifolium mean_doy
Parameter Distribution
Fixed GDD
acer circinatum F
acer rubrum F
populus tremuloides F
vaccinium parvifolium F
Parameter Distribution
Linear
acer circinatum intercept
acer circinatum slope
acer rubrum intercept
acer rubrum slope
populus tremuloides intercept
populus tremuloides slope
vaccinium parvifolium intercept
vaccinium parvifolium slope
Parameter Distribution
GDD
acer circinatum F
acer circinatum t1
acer circinatum T_base
acer rubrum F
acer rubrum t1
acer rubrum T_base
populus tremuloides F
populus tremuloides t1
populus tremuloides T_base
vaccinium parvifolium F
vaccinium parvifolium t1
vaccinium parvifolium T_base
Parameter Distribution
Harvard Forest
NPN
H.J. Andrews
mean_value
median_value

Figure S10

Figure S11: As in Figure S6, but for the Alternating and Uniforc models.

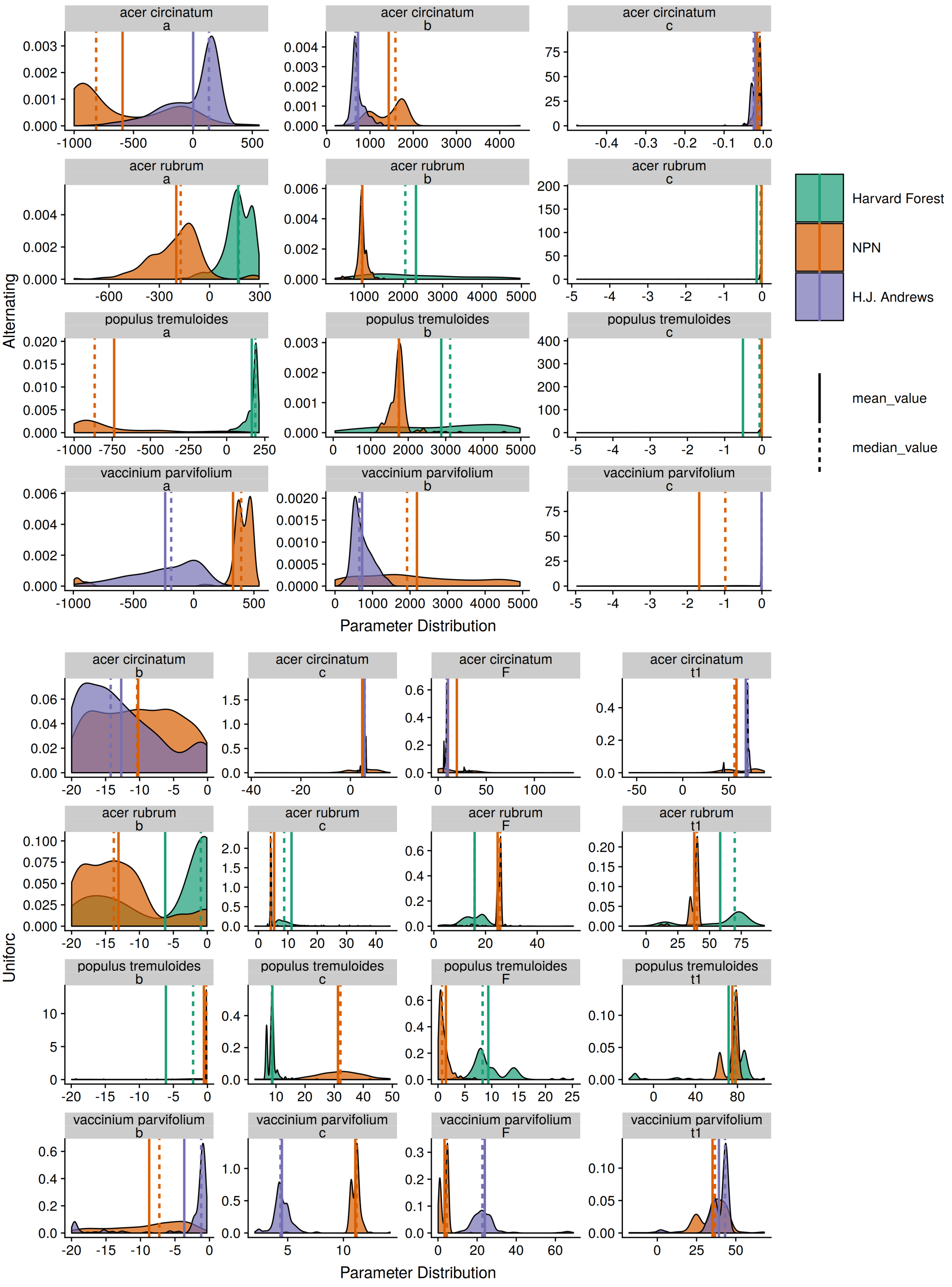
Alternating
acer circinatum a
acer circinatum b
acer circinatum c
acer rubrum a
acer rubrum b
acer rubrum c
populus tremuloides a
populus tremuloides b
populus tremuloides c
vaccinium parvifolium a
vaccinium parvifolium b
vaccinium parvifolium c
Parameter Distribution
Harvard Forest
NPN
H.J. Andrews
mean_value
median_value
Uniforc
acer circinatum b
acer circinatum c
acer circinatum F
acer circinatum t1
acer rubrum b
acer rubrum c
acer rubrum F
acer rubrum t1
populus tremuloides b
populus tremuloides c
populus tremuloides F
populus tremuloides t1
vaccinium parvifolium b
vaccinium parvifolium c
vaccinium parvifolium F
vaccinium parvifolium t1
Parameter Distribution

Figure S11

Table S1: Species used in the analysis along with the sample size from each dataset. The numbers indicate the sample size of the training data and the size of the testing data in parenthesis.

species	phenophase	phenophase_type	harvard	hjandrews	hubbard	jornada	npn
acer circinatum	371	Budburst	-	266 (66)	-	-	39 (10)
acer circinatum	501	Flowers	-	116 (29)	-	-	33 (8)
acer pensylvanicum	371	Budburst	80 (20)	-	-	-	34 (9)
acer rubrum	371	Budburst	100 (25)	-	-	-	957 (239)
acer rubrum	501	Flowers	96 (24)	-	-	-	668 (167)
acer saccharum	371	Budburst	60 (15)	-	164 (41)	-	365 (91)
betula alleghaniensis	371	Budburst	60 (15)	-	178 (44)	-	133 (33)
betula alleghaniensis	501	Flowers	26 (7)	-	-	-	64 (16)
betula lenta	371	Budburst	58 (14)	-	-	-	96 (24)
betula papyrifera	371	Budburst	76 (19)	-	-	-	96 (26)
betula papyrifera	501	Flowers	18 (5)	-	-	-	36 (9)
fagus grandifolia	371	Budburst	76 (19)	-	177 (44)	-	259 (65)
fraxinus americana	371	Budburst	84 (21)	-	-	-	90 (23)
fraxinus americana	501	Flowers	22 (6)	-	-	-	52 (13)
ilex verticillata	371	Budburst	35 (9)	-	-	-	26 (6)
larrea tridentata	501	Flowers	-	-	-	27 (7)	118 (30)
nyssa sylvatica	371	Budburst	27 (7)	-	-	-	63 (16)
pinus strobus	496	Budburst	38 (10)	-	-	-	77 (19)
populus tremuloides	371	Budburst	38 (10)	-	-	-	208 (51)
populus tremuloides	501	Flowers	17 (4)	-	-	-	79 (22)
prosopis glandulosa	501	Flowers	-	-	-	49 (12)	78 (20)
prunus serotina	371	Budburst	58 (14)	-	-	-	228 (57)
pseudotsuga menziesii	480	Budburst	-	182 (46)	-	-	38 (10)
quercus alba	371	Budburst	62 (15)	-	-	-	174 (43)
quercus rubra	371	Budburst	80 (20)	-	-	-	242 (60)
quercus rubra	501	Flowers	56 (14)	-	-	-	127 (32)
quercus velutina	371	Budburst	77 (19)	-	-	-	72 (18)
rhododendron macrophyllum	371	Budburst	-	84 (21)	-	-	48 (12)
rhododendron macrophyllum	501	Flowers	-	27 (7)	-	-	50 (12)
trillium ovatum	488	Budburst	-	222 (55)	-	-	68 (17)
trillium ovatum	501	Flowers	-	169 (42)	-	-	60 (15)
vaccinium corymbosum	371	Budburst	38 (10)	-	-	-	60 (15)
vaccinium corymbosum	501	Flowers	38 (10)	-	-	-	65 (16)
vaccinium parvifolium	371	Budburst	-	149 (37)	-	-	25 (6)
vaccinium parvifolium	501	Flowers	-	162 (41)	-	-	25 (6)

Table S1

Table S2: Overall best models when doing cross dataset comparisons. Observations for all species and phenophases were aggregated together to calculate RMSE and Pearsons coefficient for each combination of Parameter source (either USA-NPN or LTER), observation source (either USA-NPN or LTER), and model (6 possible phenology models). Bold indicates the best performing model for a specific parameter and observation combination, with some combinations having ties among multiple models.

Table S2

Parameter Source	Held Out Observation Source	Alternating		Fixed GDD		GDD		Linear		M1		MSB		Naive		Uniforc	
		p	RMSE	p	RMSE	p	RMSE	p	RMSE	p	RMSE	p	RMSE	p	RMSE	p	RMSE
LTER	LTER	0.87	8.73	0.84	10.14	**0.90**	7.89	0.81	10.27	**0.90**	7.89	0.87	8.73	0.72	12.35	**0.90**	**7.86**
LTER	USA-NPN	0.44	26.07	**0.72**	22.70	0.68	20.52	0.65	22.94	0.68	20.52	0.44	26.07	0.34	31.25	0.70	**19.69**
USA-NPN	LTER	0.63	16.17	0.69	16.15	0.70	13.73	0.68	16.59	0.71	13.79	0.66	15.79	0.61	27.08	**0.72**	**13.48**
USA-NPN	USA-NPN	0.80	15.27	0.76	20.18	**0.82**	14.67	0.77	16.19	**0.82**	14.71	0.80	15.20	0.53	21.57	**0.82**	**14.37**
